# Supplementary material for: P-TRAP: a Panicle Trait Phenotyping tool
Source: BMC Plant Biol. 2013 Aug 29;13:122. doi: 10.1186/1471-2229-13-122 (PMC3848748; doi:10.1186/1471-2229-13-122)
Supplement: Additional file 7 — P-TRAP source code. The Netbeans project that contains the source code of the application. [file 1471-2229-13-122-S7.zip › souce.zip!/P-TRAP source code/Grain/doc/javadoc/org-basmati-fileexplorer/index-all.html]

Index (File Explorer)


JavaScript is disabled on your browser.


- Package
- Class
- Tree
- Deprecated
- Index
- Help

*org.basmati.fileexplorer 1.0*

- Prev
- Next

- Frames
- No Frames

- All Classes

C G O R 


## C

createNodeDelegate() - Method in class org.basmati.fileexplorer.RiceImageDataObject


## G

getLookup() - Method in class org.basmati.fileexplorer.RiceImageDataObject


## O

org.basmati.fileexplorer - package org.basmati.fileexplorer


## R

RiceImageDataObject - Class in org.basmati.fileexplorer


RiceImageDataObject(FileObject, MultiFileLoader) - Constructor for class org.basmati.fileexplorer.RiceImageDataObject

C G O R

- Package
- Class
- Tree
- Deprecated
- Index
- Help

*org.basmati.fileexplorer 1.0*

- Prev
- Next

- Frames
- No Frames

- All Classes
